# Determining the Best Immunization Strategy for Protecting African Children Against Invasive *Salmonella* Disease

**DOI:** 10.1093/cid/ciy386

**Published:** 2018-05-09

**Authors:** Hyon Jin Jeon, Gi Deok Pak, Justin Im, Ellis Owusu-Dabo, Yaw Adu-Sarkodie, Amy Gassama Sow, Abdramane Bassiahi Soura, Nagla Gasmelseed, Karen H Keddy, Morten Bjerregaard-Andersen, Frank Konings, Abraham Aseffa, John A Crump, Yun Chon, Robert F Breiman, Se Eun Park, Ligia Maria Cruz Espinoza, Hye Jin Seo, Jürgen May, Christian G Meyer, Jason R Andrews, Ursula Panzner, Vera von Kalckreuth, Thomas F Wierzba, Raphaël Rakotozandrindrainy, Gordon Dougan, Myron M Levine, Joachim Hombach, Jerome H Kim, John D Clemens, Stephen Baker, Florian Marks

**Affiliations:** 1 International Vaccine Institute, Seoul, Republic of Korea; 2 Kumasi Center for Collaborative Research in Tropical Medicine, Kumasi, Ghana; 3 Departments of Global and International Health, Kwame Nkrumah University of Science and Technology, Kumasi, Ghana; 4 Departments of Clinical Microbiology, School of Medical Sciences, Kwame Nkrumah University of Science and Technology, Kumasi, Ghana; 5 Institute Pasteur de Dakar; 6 Université Cheikh Anta Diop de Dakar, Senegal; 7 Institut Supérieur des Sciences de la Population, University of Ouagadougou, Burkina Faso; 8 Faculty of Medicine, University of Gezira, Wad Medani, Sudan; 9 Faculty of Science, University of Hafr Al Batin, Saudi Arabia; 10 National Institute for Communicable Diseases, Johannesburg, South Africa; 11 Faculty of Health Sciences, University of the Witwatersrand, Johannesburg, South Africa; 12 Bandim Health Project, Bissau, Guinea-Bissau; 13 Research Center for Vitamins and Vaccines, Copenhagen, Denmark; 14 Armauer Hansen Research Institute, ALERT Campus, Addis Ababa, Ethiopia; 15 Kilimanjaro Christian Medical Centre, Moshi, Tanzania; 16 Division of Infectious Diseases and International Health, Duke University Medical Center; 17 Duke Global Health Institute, Duke University, Durham, North Carolina; 18 Centre for International Health, University of Otago, Dunedin, New Zealand; 19 Global Health Institute, Emory University, Atlanta, Georgia; 20 Oxford University Clinical Research Unit, Ho Chi Minh City, Vietnam; 21 Bernhard Nocht Institute for Tropical Medicine, Hamburg; 22 Institute of Tropical Medicine, Eberhard-Karls University Tübingen, Germany; 23 Duy Tan University, Da Nang, Vietnam; 24 Division of Infectious Diseases and Geographic Medicine, Stanford University, California; 25 University of Antananarivo, Madagascar; 26 Department of Medicine, University of Cambridge, United Kingdom; 27 Department of Medicine, University of Maryland School of Medicine, Baltimore; 28 World Health Organization, Geneva, Switzerland; 29 International Centre for Diarrheal Disease Research, Bangladesh, Dhaka; 30 Fielding School of Public Health, University of California, Los Angeles; 31 Korea University School of Medicine, Seoul, Republic of Korea

**Keywords:** *Salmonella* Typhi, iNTS disease, immunization, typhoid conjugate vaccine

## Abstract

**Background:**

The World Health Organization recently prequalified a typhoid conjugate vaccine (TCV), recommending its use in persons ≥6 months to 45 years residing in typhoid fever (TF)–endemic areas. We now need to consider how TCVs can have the greatest impact in the most vulnerable populations.

**Methods:**

The Typhoid Fever Surveillance in Africa Program (TSAP) was a blood culture-based surveillance of febrile patients from defined populations presenting at healthcare facilities in 10 African countries. TF and invasive non-typhoidal *Salmonella* (iNTS) disease incidences were estimated for 0–10 year-olds in one-year age increments.

**Results:**

*Salmonella* Typhi and iNTS were the most frequently isolated pathogens; 135 and 94 cases were identified, respectively. Analysis from three countries was excluded (incomplete person-years of observation (PYO) data). Thirty-seven of 123 TF cases (30.1%) and 71/90 iNTS disease cases (78.9%) occurred in children aged <5 years. No TF and 8/90 iNTS infections (8.9%) were observed in infants aged <9 months. The TF incidences (/100 000 PYO) for children aged <1 year and 1 to <2 years were 5 and 39, respectively; the highest incidence was 304 per 100 000 PYO in 4 to <5 year-olds. The iNTS disease incidence in the defined age groups ranged between 81 and 233 per 100 000 PYO, highest in 1 to <2 year-olds. TF and iNTS disease incidences were higher in West Africa.

**Conclusions:**

High burden of TF detected in young children strengthens the need for TCV introduction. Given the concurrent iNTS disease burden, development of a trivalent vaccine against *S.* Typhi, *S.* Typhimurium, and *S.* Enteritidis may be timely in this region.

## Teaser

A significant burden of typhoid fever in children aged <5 years in sub-Saharan Africa merits the introduction of typhoid conjugate vaccine at age 9 months, which coincides with the first dose of measles vaccine.

The recently published multicenter Typhoid Fever Surveillance in Africa Program (TSAP) revealed a significant burden of invasive *Salmonella* disease in sub-Saharan Africa [1]. These data largely confirmed estimates generated by two recent systematic reviews [2, 3] and suggested that the burden of typhoid fever in parts of sub-Saharan Africa is increasing [2]. Correspondingly, the relative contribution of *Salmonella* as an agent of invasive disease is rising, contrasting the dramatic reduction of invasive bacterial disease caused by *Haemophilus influenzae* type b and *Streptococcus pneumoniae* in African infants, a consequence of routine immunization against these pathogens. Such immunization programs are not yet available to prevent invasive *Salmonella* disease.

*Salmonella enterica* serovar Typhi (*S.* Typhi) causes typhoid fever, and the *S. enterica* serovar Paratyphi A (less commonly B and C) causes the clinically indistinguishable disease paratyphoid fever. Infection with other *Salmonella* serovars, the nontyphoidal *Salmonella* (NTS) serovars, generally result in a self-limiting diarrhea. However, NTS organisms can also induce a systemic infection in susceptible individuals, resulting in invasive NTS (iNTS) disease [4]. Globally, typhoid fever and iNTS disease are estimated to be responsible for 20.6 and 3.4 million illnesses and 223000 and 681000 deaths annually, respectively [3, 5–7].

Presently, there are three typhoid vaccines available: (1) an injectable polysaccharide vaccine composed solely of purified Vi antigen (ViPS vaccine), which is licensed for adults and children aged ≥2 years; (2) a live attenuated oral vaccine available in capsular formulation, licensed for adults and children aged ≥5 years (Ty21a); and (3) a recently licensed typhoid conjugate vaccine (TCV), comprising of Vi antigen covalently linked to a carrier protein. The TCV elicits sustained immunoglobulin G anti-Vi response after single-dose administration to infants, toddlers, and older age groups [8, 9].

The efficacy of the ViPS and Ty21a vaccines is approximately 70% at 3 years after immunization and about 60% for Ty21a 7 years after immunization [10]. The ViPS vaccine is safe, well tolerated, but weakly immunogenic in infants, with antibody titers being only short-lived [10–12]; Ty21a is not licensed for use in preschool children, infants, or toddlers. These particular properties, in addition to limited global production capacity, render these two vaccines ineligible for Gavi subsidy in developing countries. The World Health Organization (WHO) has recommended vaccination with a single dose of TCV in children from 6 months of age to adults up to 45 years of age living in areas where typhoid fever is endemic, with an emphasis on introduction alongside other Expanded Program on Immunization (EPI) vaccines at 9 months or in the second year of life [13].

Data have existed for approximately 20 years showing that a TCV, consisting of Vi linked to exotoxin A of *Pseudomonas aeruginosa* (Vi-rEPA), was immunogenic in preschool children and young infants (with doses given at 2, 4, and 6 months of age). Furthermore, two doses of Vi-rEPA conferred 89% protection over 46 months in 2-4-year-old Vietnamese children in a randomized controlled trial. That study generated substantial anticipation that Vi-rEPA (and other TCVs) would be licensed and prequalified in a reasonable time frame [14]. However, it was not until the end of 2017 that a TCV, Typbar-TCV®, manufactured by Bharat Biotech International and licensed by the Indian National Regulatory Agency, was recommended by the Scientific Advisory Group of Experts (SAGE) for use in infants, toddlers, and older persons [15]. In 2018, the vaccine was prequalified by the WHO, making it eligible for United Nations agencies procurement and Gavi support [16].

In a randomized, placebo-controlled human volunteer challenge study, Typbar-TCV® conferred protection against 54.6% (95% confidence interval [CI], 26.8%–71.8%) of typhoid fever infections when the primary endpoint was defined as fever of ≥38.0°C for ≥12 hours or *S*. Typhi bacteremia. Using an alternative endpoint of *S.* Typhi bacteremia after fever of ≥38.0°C, the vaccine efficacy of Typbar-TCV® was 87.1% (95% CI, 47.2%**–**96.9%), compared with 52.3% (95% CI, –4.2% to 78.2%) for ViPS for the same endpoint [17]. Phase III and IV trials are planned in endemic countries, and manufacturers are developing other TCV candidates [18, 19]. Although the TCVs are effective only against *S.* Typhi, several vaccines against iNTS disease are at an early stage of development, some of which are bivalent, targeting both *S. enterica* serovars Enteritidis and Typhimurium [20, 21]. A typhoid fever/iNTS disease conjugate vaccine targeting *S.* Enteritidis, *S.* Typhimurium, and *S.* Typhi is commencing phase 1 clinical trials in 2018 [22].

With a first TCV now available for procurement and Gavi subsidy, and with a battery of prospective vaccines in the pipeline, relevant epidemiological data are needed to inform decision making regarding vaccine target groups and introduction strategies. A detailed understanding of typhoid fever hot spots and disease in infants and young children are important for identifying the most effective vaccination strategy. Furthermore, the feasibility of introducing TCV into the existing EPI needs to be considered alongside priorities of targeting children early enough to prevent infection in infancy. Here, we reanalyze the data obtained through the TSAP to obtain incidences in the children <5 years old by yearly age increments and stratify them by different African regions.

## METHODS

To understand the occurrence of invasive *Salmonella* disease in infants and young children, we conducted further analysis using recently published TSAP disease incidence data [1], stratifying by 12-month age increments for children aged 0–10 years of age (Table 1) and by region, East versus West African TSAP study sites (Table 2). Briefly, the TSAP study performed blood culture-based surveillance in febrile persons presenting at healthcare facilities in 10 countries: Burkina Faso (2 sites), Ethiopia, Ghana, Guinea-Bissau, Kenya, Madagascar (2 sites), Senegal, South Africa, Sudan, and Tanzania (2 sites) [1, 23].

**Table 1.** Incidence of Invasive *Salmonella* Infections in Children and Adults Enrolled in the Typhoid Surveillance in Africa Program, March 2010 to January 2014^a^

Age Group, y	Patients Enrolled, No.	PYO^b^	*Salmonella* Typhi			iNTS Disease		
			Crude Cases, No.	Cases Adjusted for Recruitment, No.^c^	Adjusted Incidence per 100000 PYO (95% CI)	Crude Cases, No.	Cases Adjusted for Recruitment, No.^c^	Adjusted Incidence per 100000 PYO (95% CI)
0 to <1	1217	8658	1	1	5.4 (.6–47.3)	14 (15)^d^	33	81.4 (27.4–241.6)
1 to <2	1057	9102	4	7	39.4 (12.3–125.9)	27	77	233.2 (81.5–667.6)
2 to <3	818	6407	7	19	152.7 (56.3–413.9)	13	35	138.7 (47.0–409.2)
3 to <4	685	5507	13	27	239.6 (91.1–630.4)	15	38	206.3 (70.4–603.9)
4 to <5	575	4800	12 (16)^d^	28	304.1 (116.4–794.3)	2	8	46.2 (13.0–164.1)
5 to <6	427	13638	7	13	56.8 (20.0–161.4)	4	8	18.4 (5.3–63.8)
6 to <7	356	8277	6 (10)^d^	17	87.7 (31.9–241.7)	1	4	11.2 (2.7–46.6)
7 to <8	376	16594	11 (12)^d^	21	111.0 (41.4–297.8)	2	6	19.5 (5.5–69.3)
8 to <9	310	10047	9	16	116.3 (42.2–320.6)	1	3	12.0 (2.6–55.6)
9 to <10	254	9019	4 (5)^d^	8	70.3 (23.3–212.5)	2	6	30.8 (8.7–109.5)
10 to <15	1004	23977	26 (27)^d^	42	109.8 (43.1–279.6)	3	7	11.1 (3.1–39.2)
15 to <20	566	14283	7 (8)^d^	10	57.3 (19.4–168.9)	1	3	15.9 (3.5–73.4)
20 to <35	1465	48352	13 (16)^d^	21	38.2 (14.3–101.9)	1	4	5.2 (1.2–21.5)
≥35	1288	34576	3	3	8.8 (2.1–37.1)	4 (5)^d^	6	13.8 (3.8–50.1)
Total	10398	213241	123 (138)^d^	233		90 (92)^d^	239	

Abbreviations: CI, confidence interval; iNTS, invasive nontyphoidal *Salmonella*; PYO, person-years of observation.

^a^Ethiopia, South Africa, and Senegal were excluded from the analysis because no PYO data were available.

^b^Study population adjusted for healthcare-seeking behavior.

^c^Crude cases adjusted for recruitment proportion (No. of patients analyzed/No. with febrile illness from study area who visited a recruitment health facility × 100.)

^d^Crude cases adjusted for recruitment pattern unique to the site in Tanzania: before 11 November 2011 every fifth eligible patient was recruited, and from 11 November 2011 every second eligible patient was recruited. Adjusted cases (presented parenthetically) were used to calculate crude rates.

**Table 2.** Incidence of Invasive *Salmonella* Infections in Children and Adults, by Region (East and West), Enrolled in the Typhoid Fever Surveillance in Africa Program, March 2010 to January 2014^a^

Age Group, y	*Salmonella* Typhi Incidence/100 000 PYO		iNTS Disease Incidence/100 000 PYO	
	East African Region	West African Region	East African Region	West African Region
0–1 to <1	41.4 (5.7–300.6)	0	165.6 (61.5–446.3)	464.5 (321.5–671.3)
1 to <2	65.8 (16.2–267.2)	82.5 (34.0–200.2)	32.9 (4.5–238.7)	1253.8 (998.8–1573.9)
2 to <3	45.5 (6.3–330.6)	427.5 (267.9–682.1)	0	831.2 (594.6–1162.1)
3 to <4	205.3 (84.6–498.2)	683.7 (443.6–1053.8)	41.1 (5.7–298.1)	1204.6 (869.6–1668.7)
4 to <5	457.1 (244.2–855.6)	689.0 (431.8–1099.3)	0	267.9 (126.7–566.8)
5 to <6	51.7 (19.2–139.4)	152.4 (78.7–295.2)	12.9 (1.8–93.9)	118.6 (56.0–250.8)
6 to <7	222.3 (110.3–448.1)	192.4 (99.3–372.5)	0	85.5 (31.7–230.4)
7 to <8	67.2 (33.3–135.4)	277.4 (160.1–480.7)	0	149.4 (70.6–316.0)
8 to <9	75.9 (31.3–184.2)	318.0 (174.9–578.2)	0	86.7 (27.6–272.4)
9 to <10	63.8 (23.7–171.8)	182.0 (75.0–441.6)	0	254.8 (120.4–538.9)
10 to <15	141.8 (92.0–218.6)	228.9 (148.5–352.9)	0	76.3 (36.1–161.4)
15 to <20	104.9 (52.1–211.5)	30.0 (7.4–122.0)	0	45.1 (14.3–141.5)
20 to <35	90.7 (55.2–148.8)	16.3 (6.7–39.5)	0	13.0 (4.8–35.1)
≥35	15.6 (5.0–49.0)	0	31.2 (13.9–70.1)	0

Abbreviations: iNTS, invasive nontyphoidal *Salmonella*; PYO, person-years of observation.

^a^West African countries included Ghana, Burkina Faso, and Guinea-Bissau; East African countries, Kenya, Tanzania, Sudan, and Madagascar.

Patients of all ages (except in Ghana where only children aged <15 years were enrolled) with acute fever (≥37.5°C axillary or ≥38.0°C tympanic) or history of fever (≥3 consecutive days in the past 7 days) attending healthcare facilities in a defined catchment area were enrolled, and blood samples were taken to isolate bacterial pathogens. In total, 13431 blood samples were assessed, from which 568 were positive yielding non-contaminant isolates. The most commonly isolated non-contaminant pathogens were *S.* Typhi (135; 23.8%), iNTS (94; 16.5%), *Staphylococcus aureus* (70; 12.3%), *E. coli* (47; 8.3%), and *S. pneumoniae* (43; 7.6%); 179 other non-contaminant pathogens (31.5%) were identified in lower frequencies (ie, *Klebsiella pneumoniae* and *oxytoca*, *Acinetobacter* spp., *Enterobacter* spp., and *Pseudomonas* spp.). The proportion of contaminated blood cultures was highest in Burkina Faso reaching 24% in one site, 12% in Guinea-Bissau, and <10% in the remaining settings presented here [1]. Burkina Faso, Ghana, Guinea-Bissau represented West Africa; Kenya, Tanzania, Sudan, and Madagascar, East Africa. Ethiopia, South Africa, and Senegal were excluded, owing to the absence of person-years of observation (PYO) data from these sites.

To evaluate the disease burden across all TSAP sites, we deployed a Poisson model that was adjusted for site as a random effect. This approach was used to estimate the incidences of *S.* Typhi and iNTS disease in each of the age strata. In addition, a stratified Poisson model was fitted to estimate disease incidences in the various age groups in each region (GLIMMIX in SAS software; version 9.4; SAS Institute). This model was then used to generate pan-African and regional (East and West Africa) incidence estimates. The ethics committees of all collaborating institutions at the TSAP sites and that of the International Vaccine Institute (Seoul, Republic of Korea) approved the TSAP study protocol.

## RESULTS

**Figure 1.**
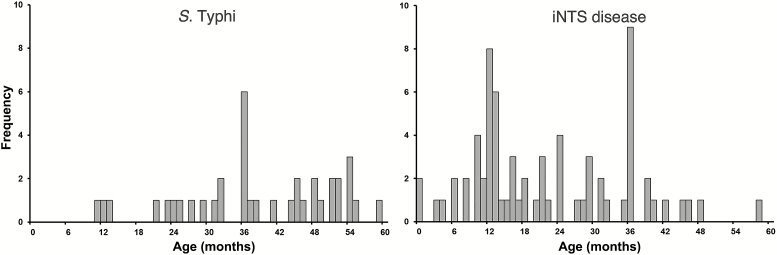
Frequency of *Salmonella* Typhi (*left*) and invasive nontyphoidal *Salmonella* (iNTS) (*right*) infections in children <5 years of age enrolled in the Typhoid Surveillance in Africa Program (TSAP) from March 2010 to January 2014.

Overall age data from all 10 countries showed *S*. Typhi and NTS to be the most frequently identified non-contaminant organisms, with a total of 135 and 94 isolations, respectively [1]. After exclusion of 12 typhoid fever and 4 iNTS disease isolates from Ethiopia, South Africa, and Senegal owing to the absence of PYO data, 37 (30.1%) of 123 cases of typhoid fever and 71 (78.9%) of 90 cases of iNTS occurred in children aged <5 years (Table 1). No cases of typhoid fever and 8 (8.9%) of 90 iNTS infections were observed in infants aged <9 months (Figure 1). Of the 37 children with typhoid fever, 17 (46.0%) were hospitalized; 58 (81.7%) of the 71 children with iNTS disease were hospitalized. This disparity in hospitalization rates was significant (*P* < .001; χ^2^-test) but did not remain significant after adjustment for age (maximum likelihood estimation; *P* = .07). Expanding these analyses to all age groups, 38 (30.9%) of 123 patients with typhoid fever and 68 of 90 (75.5%) with iNTS were hospitalized (maximum likelihood estimation; *P* = .047).

Our data demonstrated that typhoid fever was most common in children aged 2 to <8 years, whereas iNTS disease peaked in children aged <5 years, particularly in those aged <2 years (Figure 1). The annual typhoid fever incidences (per 100000 PYO) for children aged <1 year and 1 to <2 years were 5 and 39, respectively. The age group with the highest typhoid fever incidence (304/100000 PYO) was 4 to <5 years (Table 1); the age group with the highest iNTS incidence (233/100000 PYO) was 1 to <2 years (Table 1).

**Figure 2.**
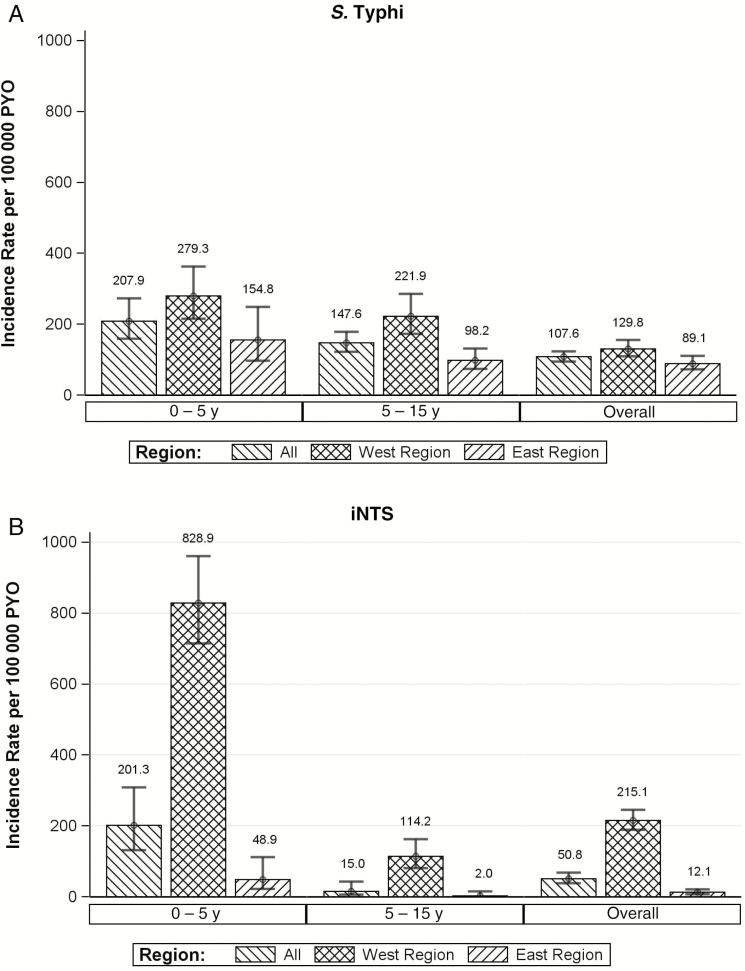
Incidence of invasive *Salmonella* infections in children by region (West and East) from the Typhoid Surveillance in Africa Program, March 2010 to January 2014. *A, Salmonella* Typhi infections. *B,* Invasive nontyphoidal *Salmonella* (iNTS) infections. West African countries included Ghana, Burkina Faso, and Guinea-Bissau; East African countries, Kenya, Tanzania, Sudan, and Madagascar. Abbreviation: PYO, person-years of observation.

After stratifying surveillance sites by geographic location (East vs West Africa) (Table 2), we observed a significant difference in disease incidence between the two regions (*P* = .009; χ^2^ test). Notably, the annual incidences of typhoid fever and iNTS disease were higher in the West African than in the East African sites (Figure 2). The cumulative incidence of typhoid fever in children aged <5 years, per 100000 PYO, was 279 in West Africa, compared with 154 in East Africa. The iNTS incidence in the same age group, per 100 000 PYO, was 828 in West and 48 in East Africa (Figure 2).

## DISCUSSION

The data evaluated here are the first systematically collected multisite incidence data for typhoid fever and iNTS in young children in Africa, which provides evidence of a substantial typhoid fever burden that merits vaccine use. Our findings on typhoid fever burden are largely consistent with recent disease burden estimates [2]. A high incidence of typhoid fever in children aged 2 to <4 years has been recorded in previous studies in Kenya [24] and India [25], and similar results have also been obtained from Vietnam [26] and Bangladesh [27]. The “Diseases of the Most Impoverished” program provided data for children <2 years of age in two countries, India and Indonesia, and found that the burden of typhoid fever in this age group was comparatively low [28]. In contrast, iNTS disease seems to affect mainly individuals in sub-Saharan Africa, and its geographic range correlates with the occurrence of malaria [29].

The delay of a WHO-prequalified TCV resulted in many years of sparse use of typhoid vaccines in endemic areas, because the existing vaccines were not eligible for Gavi subsidy and only one vaccine manufacturer had a WHO-prequalified product. The recent availability of the first WHO-prequalified TCV and the specific SAGE recommendations constitute a potential turning point. Existing vaccine policies are being reassessed, and it is paramount to identify the optimum time of administration for the effective integration of typhoid vaccines into existing vaccination programs. Based partly on the data presented here, SAGE recently recommended the use of TCVs in children aged <2 years and proposed further disease surveillance to identify high-risk areas [15].

We found that children aged <5 years bear the highest burden of typhoid fever and iNTS infections and that West African TSAP sites had a higher disease burden than East African TSAP sites. Independent of the investigated sites in both African regions, our data suggest that there is a high burden of typhoid fever in children aged <5 years, affecting children as young as 9 months of age. These findings are aligned with the recommendation to introduce TCV at 9 months [13], in order to ensure maximum protection of the at risk population and slow the increase of multidrug-resistant *Salmonella*. Coadministration of TCV with the first dose of measles vaccine at age 9 months may be a practical approach [9]. For example, our data demonstrate that a vaccine against both typhoid fever and iNTS disease administered at 9 months of age would have been delivered before 100% (all 123) of the typhoid fever infections and 95.5% (86 of 90) of the iNTS disease infections occurred in the TSAP. Therefore, even a vaccine with a lower efficacy and a shorter duration of protection would have measurable impact on disease at a young age and may allow for boosting at a later stage. (Figure 1). Given that *S*. Typhi, a pathogen restricted to humans, is now appropriate for effective prevention through vaccination at age 9 months, in accordance with recent WHO recommendations [13], TCV should be introduced in high-risk areas without further delay.

For iNTS disease, we encourage the development of iNTS conjugate vaccines targeting *S.* Typhimurium/*S.* Enteritidis serovars that could be prioritized in malaria-endemic areas of Africa or, potentially, a combinatory trivalent (*S.* Typhi/*S.* Typhimurium/*S.* Enteritidis) vaccine to comprehensively tackle the invasive *Salmonella* disease [22]. Given that about 10% of iNTS infections occur in children aged <9 months, a future iNTS vaccine may need to be introduced at an earlier EPI time point. This could potentially be coupled with maternal vaccination before childbirth, as done for other vaccines [30]; however, more data are required to substantiate this approach for iNTS disease. Consequently, the most likely scenario would be to deploy TCV for the prevention of typhoid fever at 9 months of age and pursue the development of an iNTS vaccine to be administered earlier within the current EPI program to tackle iNTS disease.

In addition to the limitations previously outlined for the TSAP study [1], it is important to address the difficulties encountered when obtaining blood samples from infants and young children. The volume of blood collected during TSAP was often lower (<1 mL) than recommended by manufacturers of analytical devices, and a higher proportion of contamination was observed in children than in adults. These issues may have reduced the ability to detect pathogenic bacteria, including both *S.* Typhi and iNTS disease. The relatively low burden of *S.* Typhi in the blood of patients with bacteremia could also result in an underestimation of *S.* Typhi in the very young age group [31]. It is also important to note that the differences observed between West and East African sites were estimated only within TSAP sites, and further studies in other regions of Africa, particularly rural settings, are required to confirm this finding.

In conclusion, this analysis of the occurrence of invasive *Salmonella* in children aged <5 years provides evidence of the high burden of typhoid fever in Africa and complements data presented at the recent SAGE meeting, much of which was reflected in a position taken by the WHO in 2018 [13, 15]. The TCVs are now recommended for use in children aged <2 years, and there is a WHO-prequalified TCV in place for use. The high burden of typhoid fever in the TSAP sites merits introduction of TCV at age 9 months, coinciding with the first measles vaccine dose. This strategy would have permitted vaccine delivery ahead of all *S.* Typhi infections identified in TSAP. The evidence presented here also highlights a significant burden of iNTS infections in children aged <5 years, a burden that will persist beyond TCV introduction until a safe and effective and iNTS vaccine is introduced in immunization programs. Considering the high incidence of typhoid fever and iNTS disease, the development and introduction of a trivalent vaccine against *S.* Typhi, *S.* Typhimurium, and *S.* Enteritidis may be a solution for comprehensively addressing the burden of invasive *Salmonella* infections in Africa.

## Notes

***Author contributions.*** F. K., J. M., V. v. K., U. P., T. F. W., J. D. C., and F. M. conceptualized the underlying TSAP study. H. J. J., G. D .P., J. I., E. O. D., Y. A. S., A. G. S., A. S. B., N. G., K. H. K., M. B. A., F. K., A. A., J. A. C., L. C. M. E., H. J. S., U. P., v. V. K., R. R., and S. B. contributed to data acquisition from various study sites. J. M., U. P., and S. B. coordinated laboratory testing of samples. H. J. J. and G. D. P. wrote the first draft of the manuscript. H. J. J., G. D. P., Y. C., J. R. A., and F. M. conducted the statistical analyses and created the tables and figures. J. I., E. O. D., Y. A. S., J. A. C., L. M. C. E., H. J. S., J. M., C. G. M., J. R. A., U. P., S. E. P., T. F. W., G. D., M. M. L., J. H., J. H. K., J. D. C., S. B., and F. M. interpreted the data and critically revised the manuscript. All authors agree to its final content.

***Acknowledgments.*** We thank Soo Young Kwon and Ji Hyun Han for administrative support.

***Disclaimer.*** The funders had no role in study design, data collection, data analysis, data interpretation, or writing of the report. The conclusions and findings presented are those of the authors and do not necessarily reflect positions or policies of the Bill & Melinda Gates Foundation or represent the decisions or policies of the WHO or the International Vaccine Institute. The principal investigator had full access to all the study data and final responsibility for the decision to submit for publication.

***Financial support.*** This study was supported by the Bill & Melinda Gates Foundation (grants OPPGH5231 and OPP1127988). The International Vaccine Institute acknowledges its donors, including the Republic of Korea and the Swedish International Development Cooperation Agency.

***Potential conflicts of interest.*** J. H. is a staff member of the WHO. H. J. J., G. D. P., J. I., Y. C., S. E. P., L. M. C. E., H. J. S., U. P., and F. M. are funded through the Bill & Melinda Gates Foundation (grants OPPGH5321 and OPP1127988). All other authors report no potential conflicts. All authors have submitted the ICMJE Form for Disclosure of Potential Conflicts of Interest. Conflicts that the editors consider relevant to the content of the manuscript have been disclosed.
